# Living with leg lymphedema: developing a novel model of quality lymphedema care for cancer survivors

**DOI:** 10.1007/s11764-020-00919-2

**Published:** 2020-07-26

**Authors:** Catharine Bowman, Devesh Oberoi, Lori Radke, George J. Francis, Linda E. Carlson

**Affiliations:** 1 Department of Oncology, Division of Psychosocial Oncology, University of Calgary, 2202-2 Street SW, Calgary, AB T2S 3C1 Canada; 2 Department of Rehabilitation Oncology, Supportive Care South, Cancer Control Alberta, 2210-2 Street SW, Calgary, AB T2S 3C3 Canada; 3 Department of Physical Medicine and Rehabilitation, Division of Clinical Neurosciences, University of Calgary, AE173D, Tom Baker Cancer Center, 1331-29 Street NW, Calgary, AB T2N 4N2 Canada

**Keywords:** Lower-extremity lymphedema, Cancer survivorship, Rehabilitation oncology, Healthcare model, Psychosocial well-being, Barriers to healthcare

## Abstract

**Purpose:**

Lower-extremity lymphedema (LEL) is a lifelong consequence of cancer therapy and can lead to serious physical and psychosocial complications for many cancer survivors. However, clinical knowledge and treatment of LEL remain minimal. The purpose of this study was to integrate perspectives of lymphedema patients and healthcare providers (HCPs) on LEL to develop a novel model for quality lymphedema care.

**Methods:**

A mixed-methods approach was implemented. Standardized questionnaires and semi-structured interviews were used to assess psychosocial well-being and experiences of LEL patients. Interviews were also used to evaluate the clinical experiences of HCPs working within tumour groups associated with cancer-related LEL. Thematic analysis was used to analyse qualitative data.

**Results:**

Twenty-two patients and eleven HCPs participated in this study. Patient QOL, generalized anxiety and depressive symptom scores revealed a complex interplay between psychosocial well-being and supportive LEL care after cancer. Three themes emerged from interviews with patients (*n* = 19) and HCPs (*n* = 11): level of lymphedema knowledge, effectiveness of rehabilitation oncology services and barriers to care.

**Implications for Cancer Survivors:**

We developed a novel model for quality lymphedema care that emphasizes the importance of continued physical and psychosocial support for LEL patients, while illustrating the importance of HCPs in facilitating a smooth transition for patients to LEL care after cancer treatment.

**Electronic supplementary material:**

The online version of this article (10.1007/s11764-020-00919-2) contains supplementary material, which is available to authorized users.

## Introduction

Lymphedema is a chronic lymphatic disease, affecting more than 200 million individuals worldwide [1]. In North America, lymphedema is commonly caused by cancer treatment, leaving many patients with lifelong discomfort, pain and impaired physical functioning. Further, treatment-associated damage to the lymphatic system can lead to severe infection or cancer recurrence, diminishing patients’ quality of life (QOL) after cancer [2–4].

Lymphedema management relies upon time-consuming and physically demanding physical therapies administered by both healthcare professionals and patients themselves. Therapies include specialized skin care, compression, exercise and manual lymphatic drainage (MLD) [5]. To further augment therapeutic challenges, treatment costs are not fully covered by health insurance or public healthcare in North America, leaving patients to fund lymphedema care out-of-pocket [5,6]. Despite the availability of diagnostic guidelines, patients often receive inconsistent diagnoses and lymphedema-related information following cancer treatment [5,7]. Brown et al. [8] reported 78% of lower-extremity cancer survivors reported being unsure of or never being told about lower-extremity lymphedema (LEL) following cancer treatment. Considering that cancer patients from a variety of tumour groups are at a lifelong increased risk for lymphedema, it is crucial that diagnostic tools and lymphedema knowledge are effectively used within oncology outpatient services. The challenge of lymphedema knowledge dissemination and diagnosis is multi-factorial. Healthcare providers (HCPs) often receive minimal training on lymphatic pathologies during medical education and thus may not have the necessary resources and information needed to address lymphedema in clinic [9,10]. These challenges are augmented by issues associated with lymphedema tracking and continuity of care beyond active cancer treatment [9]. Consequently, patients may receive delayed lymphedema treatment, if any.

Current literature heavily focuses upon the psychosocial manifestations of breast cancer-related arm lymphedema [11]. However, up to 47% of patients who have undergone treatment for gynaecological cancer may later develop LEL [12]. Melanoma, prostate and penile cancers are also associated with LEL development, leaving many cancer-related lymphedema experiences minimally understood [13,14]. Research on LEL is limited but demonstrates the adverse physical, emotional, social and familial impact of LEL on patients. Considering the life-changing circumstances that patients have already experienced due to cancer therapy as well as the consequences of untreated lymphedema, it is crucial that all tumour groups at-risk for LEL are considered in order to develop the holistic healthcare infrastructure required to care for LEL patients during and beyond their active cancer treatment.

The aim of this study was to integrate the perspectives of lymphedema patients and HCPs on LEL to develop a novel model for quality lymphedema care. The objectives were to explore perspectives on (1) knowledge of lymphedema, (2) the effectiveness of rehabilitation oncology services for lymphedema management and (3) barriers to lymphedema care. These perspectives were amalgamated to develop the proposed model of care.

## Methods

### Study design

A mixed-methods approach was used. Quantitative and qualitative methods explored the psychosocial well-being and experiences of LEL patients, using standardized questionnaires and semi-structured interviews, respectively. Interviews were further used to evaluate the clinical experiences of HCPs working within tumour groups associated with cancer-related LEL. The study was approved by The Health Research Ethics Board of Alberta-Cancer Committee (#HREBA-CC-18-0398).

### Participants and setting

Participants were recruited across two populations: patients and HCPs.

#### Patients

##### Inclusion criteria

Patient participants were included if they had received a diagnosis of cancer-related LEL at least 1 month after completing active cancer treatment. There was no upper threshold for length of time since diagnosis. All cancer types were considered, provided that the patient’s LEL was related to their cancer treatment. Individuals on maintenance therapies were included.

##### Exclusion criteria

Patients undergoing primary cancer treatment or experiencing active cancer were excluded from the study. As a minimum, participants must have been 1 month out of active cancer treatment.

##### Sample

Convenience sampling was used to recruit participants due to challenges associated with identifying clinically confirmed cases of lymphedema. Participants who consented to individual interviews were included in the qualitative analysis.

##### Recruitment

Primary recruitment occurred through networks at the Rehabilitation Oncology Department, Tom Baker Cancer Centre (TBCC), where the majority of regional cancer patients receive lymphedema care after active cancer treatment (Calgary, AB). TBCC staff shared study information through word-of-mouth. Consent to contact forms were provided to interested patients and the research team contacted these individuals via email. This protocol was also implemented at the Cross Cancer Institute (Edmonton, AB). The Alberta Lymphedema Association (ALA) also aided in recruitment. Study information was shared with ALA members via email and organizational social media posts. Further, potential participants were identified through the Rehabilitation Oncology Department using the electronic medical record (EMR; ARIA-MO). Invitations to contact the research team were mailed to potentially eligible individuals, who then contacted the team for further study information. Recruitment took place from October 2018 until February 2019.

#### Healthcare providers

##### Inclusion criteria

Healthcare practitioners (i.e. clinicians, need to be more specific type of clinians nurses) working within melanoma, metastatic prostate, and gynaecological tumour groups were eligible for participation. Participants required the ability to refer or aid in the referral of patients to lymphedema clinics within their clinical role.

##### Exclusion criteria

No specific exclusion criteria were outlined.

##### Sample

Convenience sampling was used to recruit HCPs across the specified tumour groups.

##### Recruitment

An informative presentation was given to tumour groups at the TBCC during morning rounds and interested HCPs’ contact information was collected. Study information was then sent to interested individuals and in-person or telephone interviews were arranged via email.

### Procedures

#### Patients

Following informed consent, an online questionnaire link was emailed to participants using a secure, web-based form designed to support data collection for research studies through the Research Electronic Data Capture (REDCap) system. A sub-set of participants completed in-person or telephone semi-structured interviews.

##### Quantitative measures

Data were obtained using validated standardized instruments. Standardized scoring guidelines were used to interpret scores for the Lymphedema Quality of Life (LYMQOL-leg) tool, Functional Assessment of Cancer Therapy-General (FACT-G), Generalized Anxiety Disorder (GAD-7) tool and the Patient Health Questionnaire (PHQ-8), as described in Table 1 [15–19].

**Table 1:** Descriptions of quantitative instruments used within the study to assess quality of life, generalized anxiety and depressive symptoms

Instrument	Description and Cronbach’s alpha Score
Lymphedema Quality of Life (LYMQOL-leg) Tool	This 27-question survey evaluates the impact of LEL on QOL. Question domains included symptoms, appearance, function and mood. Overall QOL scores are reported in this paper, where higher scores indicate greater QOL (*α* = 0.83–0.88) [15].
Functional Assessment of Cancer Therapy-General (FACT-G)	The FACT-G is a 27-item questionnaire, which evaluates QOL of those experiencing chronic illness [16]. The FACT-G is segmented into four domains: physical, social/family, emotional, and functional well-being, and there is a total score. Higher scores indicate better QOL (*α* = 0.89) [17].
Generalized Anxiety Disorder (GAD-7)	This 7-item questionnaire assesses the level of generalized anxiety experienced by patients. Higher scores indicate increased anxiety (*α* = 0.89) [18].
Patient Health Questionnaire (PHQ-8)	This 8-item questionnaire evaluates severity of depression. Higher values indicate increased frequency of depressive symptoms. A score of ≥ 10 is indicative of a high level of depressive symptomatology (*α* = 0.87) [19].

##### Qualitative semi-structured interviews

Interview topics included past and current QOL, LEL diagnosis, management strategies, changes to daily activities, clinical resource accessibility and patients’ lymphedema-related knowledge. The interview guide (Online Resource 1) was prepared through an analysis of qualitative lymphedema literature and clinical experience. In collaboration with the CancerControl Alberta Patient and Family Advisory Council and research team networks, two LEL patients were identified and took part in advisory discussions with the study coordinator (CB) to validate and refine the guide. As a secondary validation, a “trial interview” was conducted with a LEL patient not participating in the study using the patient-revised guide.

#### Healthcare providers

After providing consent, participants completed a telephone or in-person interview with the study coordinator, dependent upon participant preference. Topics included referral practices, lymphedema knowledge and education, clinical resource accessibility and strategies to improve LEL care. Similar methodology used to develop the patient interview guide (Online Resource 2) was applied to HCP guide development. Two clinicians then took part in digital advisory discussions with the study coordinator (CB) to validate and refine the interview guide.

### Analysis

A complementarity (quant. + QUAL.) mixed-methods approach was implemented for data analysis. Quantitative data were collected prior to semi-structured interviews and were used for a non-dominant analysis to validate qualitative results.

#### Quantitative

Data were initially collected and stored within REDCap and were then de-identified and transferred to SPSS V25.0 for analysis. Descriptive statistics were used to summarize demographic and disease-related characteristics. Comparisons to published norms were made on levels of anxiety, depression and QOL relative to general, cancer and lymphedema populations, where applicable.

#### Qualitative

Interviews were audio recorded and transcribed verbatim. Inductive thematic analysis was used. The study coordinator (CB) read each transcript three times to identify preliminary themes. Emergent themes were summarized and discussed with another study team member with expertise in qualitative research (DO) to establish themes. The transcript text was then coded and categorized into the established themes. An additional discussion then took place to evaluate themes within the context of coded data. Themes that failed to reach consensus were revised and a secondary analysis was undertaken.

## Results

### Participant demographics

#### Patients

Thirty-three interested individuals contacted the study team and were screened for eligibility. Twenty-five eligible individuals contacted the study team and provided consent. Reasons for ineligibility included non-cancer-related lymphedema diagnoses and inability to complete the online questionnaire. Quantitative data were collected from 22 participants and semi-structured interviews were conducted with 19 individuals. The mean age of patient participants was 60 ± 12.9 years. The majority [16, (73%)] were female and the largest group had previously experienced melanoma [9, (39%)]. Most participants resided in metropolitan areas [16, (73%)], and all participants were Caucasian [22, (100%)] (Table 2).

**Table 2:** Demographic characteristics of all participants and participants who took part in semi-structured interviews

	**All participants**		**Interviewed participants**	
	**Patients (*n*** **= 22)**	**Healthcare providers (*n*** **= 11)**	**Patients (*n*** **= 19)**	**Healthcare providers (*n*** **= 11)**
Demographic characteristic	Value (*N*, %)	Value (*N*, %)		
Sex				
Male	6, 27.3	6, 55.0	6, 31.6	6, 55.0
Female	16, 72.7	5, 45.0	13, 68.4	5, 45.0
Age				
18–29	0,0	0, 0	0, 0	0, 0
30–39	2, 9.1	2, 18.2	2, 10.5	2, 18.2
40–49	4, 18.2	5, 45.4	3, 15.8	5, 45.4
50–59	3, 13.6	2, 18.2	3, 15.8	2, 18.2
60–69	8, 36.4	2, 18.2	8, 42.1	2, 18.2
70–79	4, 18.2		3, 15.8	
80–89	1, 4.5		0, 0	
	**Mean (SD)**	**Mean (SD)**	**Mean (SD)**	**Mean (SD)**
	60.1 years (12.9)	50.8 (10.3)	59.7 years (12.5)	50.8 (10.3)
Tumour group				
Melanoma	9, 41.0	2, 18.2	7, 36.8	2, 18.2
Gynaecology	7, 31.8	7, 63.6	6, 31.6	7, 63.6
Prostate	3, 13.6	2, 18.2	3, 15.8	2, 18.2
Other	3, 13.6	0, 0	3, 15.8	0, 0
Profession				
Nurse		3, 27.2		3, 27.2
Oncologist		5, 45.5		5, 45.5
Clinical fellow		2, 18.2		2, 18.2
Other		1, 9.1		1, 9.1
Marital status				
Single	1, 4.55		1, 5.3	
Married	13, 59.1		12, 63.1	
Common-law	3, 13.6		3, 15.8	
Divorced	4, 18.2		2, 10.5	
Widower	1, 4.55		1, 5.3	
Highest level of education				
Elementary to high school	3, 13.65		2, 10.5	
Trade school	5, 22.7		5, 26.3	
Community college	7, 31.8		6, 31.6	
University (undergraduate)	4, 18.2		4, 21.1	
University (professional/post-graduate/doctoral)	3, 13.65		2, 10.5	
Area of residence	Value (*N*, %)		Value (*N*, %)	
Metropolitan	16, 72.7		13, 68.4	
Regional/remote	6, 27.3		6, 31.6	
Family income (pre-tax) CAD				
< 30,000	2, 9.1		1, 5.3	
30,000–50,000	6, 27.3		5, 26.3	
51,000–80,000	1, 4.5		1, 5.3	
81,000–120,000	2, 9.1		2, 10.5	
> 120,000	6, 27.3		5, 26.3	
Prefer not to say	5, 22.7		5, 26.3	
Area of residence				
Metropolitan	16, 72.7		13, 68.4	
Regional/remote	6, 27.3		6, 31.6	
Private health insurance				
Yes (employer sponsored/self-sponsored)	14, 63.6		12, 63.2	
No	8, 36.4		7, 36.8	

#### Healthcare providers

Eleven practitioners provided consent and participated in interviews. The mean age of participants was 50.75 ± 10.33 years (Table 2). The majority [6, (55%)] were male and represented the gynaecology tumour group [7, (64%)]. Participants were primarily oncologists [5, (45%)] and nurses [3, (27%)].

### Quantitative outcomes

#### Patients

Overall LYMQOL scores (Table 3) were similar to published norms for the Canadian lymphedema population (6.59 vs. 6.20, *P* > 0.05) [22]. QOL was also assessed using the FACT-G. Overall and domain-specific scores were significantly lower than general population norms and the Canadian lymphedema population (*P* < 0.05), indicating lower QOL, except within the social domain, where scores did not differ significantly (*P* > 0.05) [23–25]. The mean generalized anxiety score (3.77) did not differ significantly to published norms for the general population and other cancer survivors [20,21]. Finally, 13.6% of participants scored above the PHQ-8 cut-off for high depressive symptomology, which was significantly greater than the general population (8.56%, *P* < 0.05) [19].

**Table 3:** Quantitative outcomes within the patient participant group (*n* = 22)

Measurement tool	Sample mean (SD)	Cancer survivor population mean	Lymphedema population mean	General population mean	*P* value
GAD-7	3.77 (2.2)	3.33 ^[20^^]^	--	2.97 ^[21^^]^	ns
LYMQOL total	6.59 (2.2)	--	6.20 ^[22^^]^	--	ns
FACT-G total	46 (7.3)	88.5 ^[23^^]^	51.0 ^[24^^]^	80.1 ^[25^^]^	*P* < 0.01
Functional	14.77 (6.1)	--	--	18.5	*P* < 0.01
Emotional	7.45 (3.2)	--	--	19.9	*P* < 0.01
Social	17.1 (5.6)	--	--	19.1	ns
Physical	6.23 (5.1)	--		22.7	*P* < 0.01
PHQ-8	5.27 (6.0)	4.0 ^[26^^]^	--		ns
Score ≥ 10	Value (%) 13.63			Value (%) 8.56 ^[19^^]^	*P* < 0.05

### Quality care themes

**Fig. 1:**
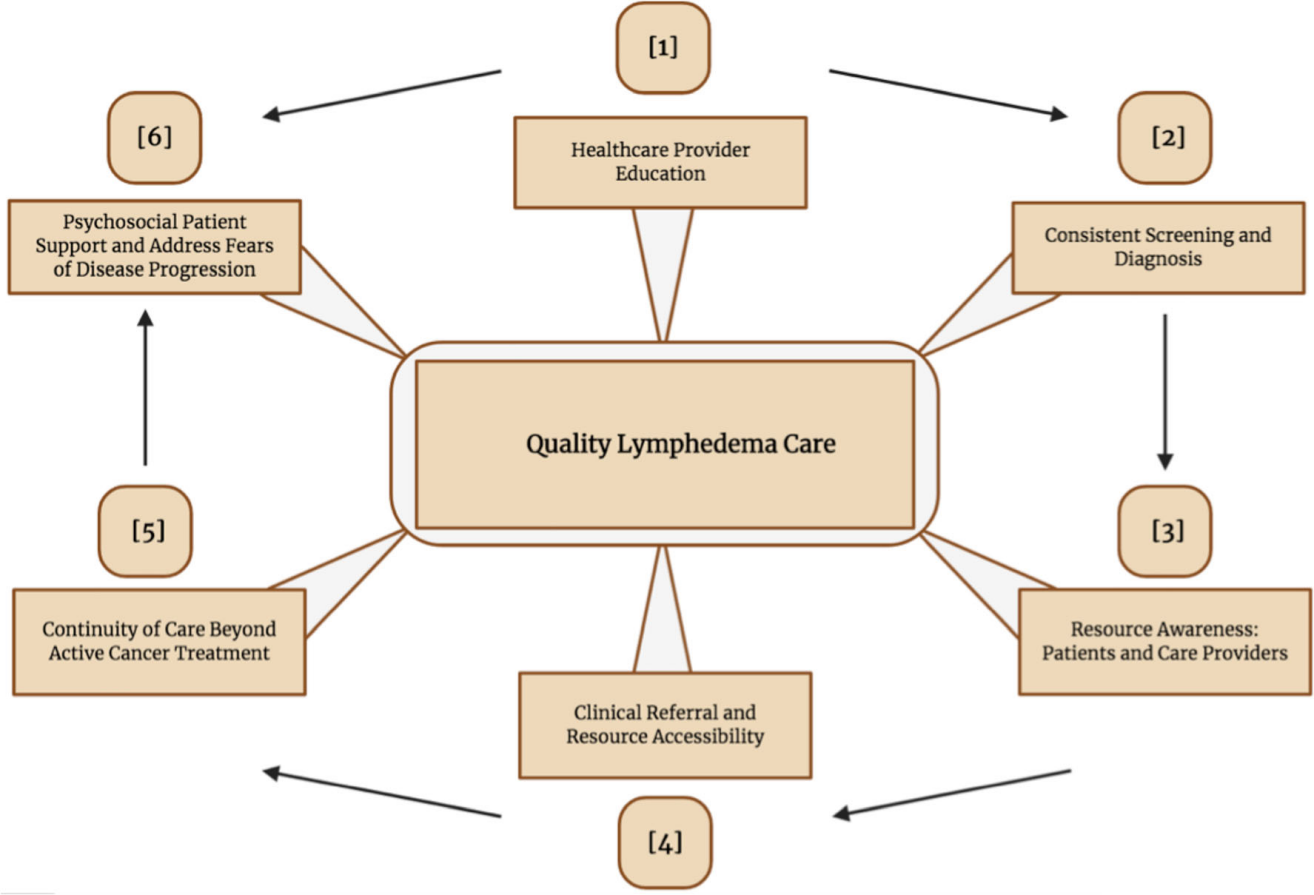
The quality lymphedema care model developed based upon perspectives shared by patient and healthcare provider participants during qualitative interviews

Results are presented separately through the perspectives of patients and HCPs, as they relate to each study objective. Subsequently, objectives across patients and providers were combined into higher-order concepts to develop a quality of care model for LEL (Fig. 1).

#### Patients

(i) Level of lymphedema knowledge
  The majority of patient participants (*n* = 16) felt they had a good understanding of lymphedema; however, few recalled receiving lymphedema education from their HCPs after cancer treatment (*n* = 4). Rather, most participants used online resources to learn about lymphedema (*n* = 11). All patients described an interest in learning more about lymphedema, particularly within the context of new research, treatment options and pathogenesis (*n* = 19). Patients described the effect that unpredictability and lack of knowledge surrounding lymphedema pathogenesis has on their fear of cancer recurrence and lymphedema progression (*n* = 13).
  “There is always a doubt… This lymphedema is not going to get better, and most things don’t stay the same. So that, by extension, means you’re probably going to get worse. So what’s that going to mean? Am I going have to start walking with crutches or cane, a walker? Am I going to be in a wheelchair? … Am I going to die from it?”
  –ID010, Age 79, Male
(ii) The effectiveness of rehabilitation oncology services for lymphedema management
  Participants addressed the benefits of accessing the Rehabilitation Oncology Clinic for initial lymphedema management, education and self-care teaching (*n* = 18). Participants discussed services provided at the clinic, which included preliminary compression bandaging, limb volume measurements and tracking and self-care instruction. The majority of participants (*n* = 14) described positive interactions with clinical personnel; however, challenges associated with clinic volume, including wait times, were also discussed (*n* = 10). One participant cited the importance of the lymphedema clinic within the context of mobility and daily activities:
  “… If I didn't do what I did, I don't think I would be walking because prior to going to see [Rehabilitation Oncology]…I couldn't grocery shop. We would go to a market … and I would sit up front and my husband would go and get what we needed…”
  –ID005, Age 59, Female
  Participants described the importance of lymphedema clinical staff for education and long-term support following active cancer care. The majority of participants (*n* = 16) accessed clinical services most heavily after referral, transitioning into self-management once their lymphedema was considered ‘under control’. These patients continued to access the clinic on an annual basis for check-ups and during exacerbations.
(iii) Barriers to lymphedema 

##### Delayed diagnosis

Delayed lymphedema diagnosis was the most commonly cited barrier to adequate care (*n* = 15). The majority of patients (*n* = 15) did not receive a timely diagnosis. Rather, participants began to “self-diagnose” through self-driven information gathering until clinical confirmation was received (*n* = 7). Participants discussed visiting multiple specialists about their lymphedema symptoms without receiving an official diagnosis, suggesting a broad expanse of lymphedema educational and diagnostic disparities that exist in the healthcare system.

“The oncologist didn’t know, the vein doctor didn’t know, my GP certainly didn’t know what was going on. Nobody knew… I just wished some of the medical people would have known about it”. – ID007, Age 72, Female

##### Costs

Several patients discussed the financial burden of lymphedema management as a barrier to receiving adequate care (*n* = 10). In some cases, patients were unable to comply with basic compression therapies due to inhibitory costs, despite having partial government subsidy (*n* = 4). Other care modalities, such as long-term MLD, are not covered by provincial healthcare. Thus, patients described the impact of paying for treatment out-of-pocket to comply with their basic care modalities:

“My MLDs, I go for once a week which are $100 a pop. That adds up. My pump was $10,000. The compression, I mean you need to keep buying it again and again to make sure it works. So, it's all expensive. There’s nothing cheap about it.” –ID005, Age 59, Female

##### Lack of awareness of lymphedema clinic

Lack of awareness of the lymphedema clinic was also cited as a barrier to symptom management, emphasizing the importance of clinical programs in preparing lymphedema patients for long-term self-care (*n* = 14). Participants shared the importance of HCP knowledge not only for care accessibility, but also for basic healthcare support:

“My [GP]… told me there was no such thing as lymphedema, it was all in my head. And while she was berating me about it, I got up and left.” –ID005, Age 59, Female

#### Healthcare providers

(i) Level of lymphedema knowledge
  All HCPs were aware of lymphedema and able to describe signs and symptoms, potential lymphedema screening strategies and referral practices (*n* = 11). The majority of HCPs did not receive formalized teaching on lymphedema during undergraduate medical education (*n* = 7). Rather, practitioners received experiential education through clinical rotations (*n* = 6).
  Participants indicated lymphedema treatment, patient resources and standardized screening protocols as three areas of education that would be beneficial in improving clinical lymphedema care. Practitioners also suggested several strategies to deliver accessible lymphedema education to HCPs working within cancer clinics. HCPs identified the importance of lymphedema resource knowledge, particularly when navigating treatment and medical supply options for patients (*n* = 8). Although one practitioner did not desire additional lymphedema education, the majority indicated interest in further education (*n* = 10). The importance and need for HCP education were summarized by one participant, who envisioned the role of education in facilitating interdisciplinary survivorship care through increased resource awareness:
  “I think that education is powerful, and especially when your patients look to you as the experts… even if you don’t know how to treat it, even if you don’t know how to help, sending them to the right resources is the most powerful thing you can do for your patient.” –HCP002, Age 53, Female
(ii) The effectiveness of rehabilitation oncology services for lymphedema management
  HCPs described the importance of the lymphedema clinic in providing adequate long-term support for patients; however, several practitioners were not familiar with the specific resources and services provided within the clinic. Some participants described screening for lymphedema regularly during post-treatment visits (*n* = 5); however, others felt that screening was not conducted enough, leading to low levels of referral to the Rehabilitation Oncology Clinic (*n* = 5). One HCP voiced concern about the clinical perception of lymphedema and its implications for referral:
  “I do believe that the culture, in general, is that there is not much we can do about [lymphedema]… You know, it is only when the patient really makes a thing about it that we finally do something and I think that’s sad because we lack knowledge here.” –HCP002, Age 53, Female
  Healthcare participants discussed individual referral protocols. Some participants used patient discomfort as an indicator for referral (*n* = 6), whereas others used physical symptomology (*n* = 5). The majority of HCPs voiced the importance of consistent screening and referral protocols in optimizing lymphedema clinic use (*n* = 8).
(iii) Barriers to lymphedema care
  Despite the need for lymphedema clinical support voiced by participants, barriers associated with facilitating a smooth transition from cancer care to rehabilitation oncology services were also discussed. Similarly to patient participants, HCPs discussed inconsistent lymphedema diagnosis as a barrier to LEL care (*n* = 6). HCPs suggested the importance of developing efficient screening tools to expedite diagnosis and treatment.
  “[Thinking] up two or three simple questions that could be added to an assessment sheet might lead to an earlier diagnosis and ultimately, treatment of lymphedema.”
  – HCP005, Age 46, Male
  Other barriers included the variable timeline associated with lymphedema onset (*n* = 3), lack of prophylactic referral protocols (*n* = 2) and varying patient follow-up after cancer treatment (*n* = 3). Practitioners discussed increased cross-departmental communication as a key factor in improving care access and continuity (*n* = 5).

### Quality lymphedema care model

Perspectives on LEL care from patients and HCPs were integrated to create a quality lymphedema care model (Fig. 1). Model branches were based upon themes that emerged from qualitative interviews.

As demonstrated within the model, a primary factor influencing lymphedema care surrounds HCP education and knowledge. The importance of this factor is two-fold, contributing to the second and sixth branches of the model, which reflect the timely diagnosis of lymphedema to facilitate early treatment and the importance of addressing fears of lymphedema progression through psychosocial support. Although several patient participants did not receive a timely diagnosis for their lymphedema, many of these patients developed their symptoms prior to the formal establishment of provincial lymphedema clinics in Alberta (*n* = 15). Thus, through increased practitioner awareness and the establishment of formalized cancer-related lymphedema programs, the potential to facilitate timely LEL diagnosis exists.

The second aspect of HCP knowledge that proved crucial to quality lymphedema care is explored within the sixth branch of the care model: the provision of holistic psychosocial support. Several patients discussed the importance of HCP knowledge in addressing their physical symptoms and psychosocial stressors (*n* = 13). Although the majority of patients (*n* = 10) did not directly express that lymphedema had a substantial impact upon their psycho-emotional well-being, participants described isolation and uncertainty as sources of lymphedema-related stress (*n* = 13). Hence, knowledge of lymphedema within oncological practice is not only critical in facilitating long-term physical symptom care, but may also aid in alleviating patients’ psychosocial stressors.

The third branch of our care model explores LEL resource awareness. All HCPs were able to describe some resources available for lymphedema patients (*n* = 11); however, the majority of participants (*n* = 10) were interested in receiving additional resource education, which was also expressed by patients. One HCP suggested the development of a lymphedema peer support program to increase resource awareness. The fourth and fifth branches of the care model are interconnected, reflecting the importance of lymphedema clinic referrals to facilitate continued supportive care. Participants described the benefits of clinical support, especially at the time of diagnosis and during complications (*n* = 18); however, high clinic volume was identified as a management barrier and area to improve clinical care.

## Discussion

This work is the first study conducted to explore the perspectives of both patients and HCPs on cancer-related LEL within a publicly funded lymphedema rehabilitation program. Perspectives of LEL patients and HCPs were integrated across three domains: lymphedema knowledge, effectiveness of rehabilitation oncology services and barriers to care. Through a secondary analysis, patient QOL, generalized anxiety and depressive symptoms were quantitatively assessed. Findings were integrated to create a model for quality lymphedema care.

Quantitative findings indicated that patients maintained similar lymphedema-specific QOL levels to published norms for the Canadian lymphedema population. However, general QOL scores were significantly lower than the general population and the Canadian lymphedema population. The mean generalized anxiety score did not differ significantly to published norms for the general population and other cancer survivors. Finally, our participants had significantly higher depressive symptomology than the general population. These quantitative data did not complement qualitative findings that suggested patients felt lymphedema did not have a direct impact upon their distress. It may be that participants had lived with LEL for a long time and therefore, established a “new normal” level of function, which although somewhat lower than otherwise healthy individuals, was satisfactory to them. Considering that participants described fear of disease progression and isolation, they may have downplayed their distress during interviews. This hypothesis aligns with published theories that suggest individuals experiencing chronic illness may suppress psycho-emotional experiences to cope with the long-term implications of their condition [27]. Further research should be conducted to elucidate the complex interplay between qualitative and quantitative findings.

Three themes emerged from qualitative studies. The first theme, level of lymphedema knowledge, was used to create two branches of the lymphedema care model. The first branch surrounded the importance of HCP knowledge and education. This is critical for optimal healthcare delivery for two reasons: timely diagnosis (branch two) and the need for psychosocial patient support (branch six). Literature indicates that early intervention and detection of subclinical lymphedema improve treatment efficacy and may prevent lymphedema exacerbations [28]. The majority of patient participants did not receive a timely diagnosis, which could be due to having developed lymphedema prior to the establishment of provincial clinics. These findings aligned with published literature that indicates poor lymphedema diagnostic rates within Canada, where few provinces have publicly funded clinical lymphedema care [29]. It is therefore speculated that the establishment of formalized lymphedema services could create the individualized medical domain needed to effectively address lymphedema and provide effective clinical care.

Furthermore, the provision of holistic psychosocial patient support (branch six) is critical in facilitating quality lymphedema care. Several patients discussed the importance of lymphedema knowledge in facilitating physical symptom management after active cancer treatment, but also in alleviating psychosocial distress through continued clinical education. These findings align with other survivorship care models, which suggest the importance of continued patient-HCP communication when navigating psychosocial survivorship concerns [30,31].

The third branch of our care model is based upon the effectiveness of rehabilitation oncology services theme. All participants described the critical role of rehabilitation oncology services in facilitating continued care for LEL patients after active cancer treatment and the need for additional resource education immediately after LEL diagnosis. Through increased resource awareness, patients may have better access to clinical supports, which must be provided long term to achieve adequate lymphedema management. As a method through which increased resource awareness for patients and HCPs may be achieved, one HCP suggested the implementation of a lymphedema peer support program. Several peer support programs have been established for cancer survivors and have been described as a potential tool for lymphedema education within literature [32,33]. However, lymphedema-specific peer programs are currently limited due to a lack of formal peer support accessibility and training in practical problem-solving for patient facilitators.

The fourth and fifth branches of the lymphedema care model are based upon the final emergent study theme: barriers to lymphedema care. Although participants described the benefits of clinical support, high clinic volume was identified as a barrier to accessing care. This finding is two-fold, emphasizing the high demand for clinical lymphedema support, while also suggesting a need for additional, adapted and/or more efficient care programs to distribute patient volume.

### Limitations

Convenience sampling was used to identify potential patient participants, and thus, demographic diversity was not optimized across participants. Considering that all participants were Caucasian and the majority of patients were older adults, it is critical to explore experiences of individuals from differing ethnic and socioeconomic backgrounds and ages to build truly accessible LEL care programs. Furthermore, many patient participants resided in metropolitan areas. Consequently, the experiences of patients in rural regions, where few lymphedema therapists and resources exist, have not been reported within this work. Future studies should explore the experiences of LEL patients living outside of urban centres to address potential challenges associated with transportation and lack of healthcare access, as reported by other lymphedema patients and cancer survivors [34–36]. Available studies on barriers to lymphedema and survivorship care access in rural settings have reported distance and travel costs, lack of trusted information access (digital and/or healthcare provider) and the familial burden of travelling for treatment as barriers to accessing adequate medical care [34,35]. As a result, patients are often left with unmet medical needs, psychological and psychosocial burden and little financial support for follow-up care and rehabilitation services [34–38]. Therefore, it is critical that future studies explore the unique barriers faced by LEL patients in rural settings in order to develop accessible models for lymphedema care after cancer. Patients were also mostly recruited through lymphedema services, representing a population who received supportive care. We suspect that the experiences of patients who had not been referred to or sought specialized care for lymphedema treatment are much worse than those in our sample, given the barriers and feelings of isolation noted by participants before receiving clinical support. Finally, all HCPs in this study practiced within an urban cancer centre, which shares close departmental connections with clinical lymphedema services. Other studies have suggested the crucial role played by community practitioners in identifying and monitoring lymphedema long term [39,40]. Therefore, further studies should be conducted to explore the experiences of community care providers to inform future lymphedema program development.

With the increased use of telehealth in cancer survivorship care, it is important to recognize that our LEL model provides insight into six aspects of care that are critical for quality lymphedema management after cancer. All branches of this care model can be directly incorporated into a telehealth platform in order to create accessible and holistic care for LEL patients after cancer treatment. Through digital patient consultation, healthcare providers can undertake preliminary assessments for diagnoses, education and treatment planning for lymphedema patients. This platform could incorporate recently developed 3D scan technologies that assess changes in lymphedematous limb volume using iPad scan acquisition, facilitating digital diagnoses that are based upon both patient history and the physical limb assessments typically undertaken in clinic [41]. Furthermore, patient and healthcare provider education and communications can be facilitated through LEL education technologies, whether a digital application or videoconference-based platform, to guide self-management techniques, such as self-massage and LEL-specific exercises. Resource awareness and accessibility can further be supported by telehealth. Information regarding lymphedema certified therapists can be provided to patients based upon geographic location, similar to the GPS-based application developed by the American Lymphedema Framework Project [42]. This form of telehealth not only includes education for patients, HCPs and therapists on lymphedema resource accessibility, but also allows patients travelling or living in rural settings to locate lymphedema therapists closest to their location. Collectively, our survivorship model incorporates several branches of care that can be directly applied to telehealth technologies. In turn, cancer survivors can access continued support after cancer treatment that addresses physical symptom care and unmet psychosocial needs.

## Conclusion

This study revealed unique findings regarding patient and HCP perspectives on LEL, demonstrating the importance of continued clinical support for cancer survivors who develop LEL. We developed a novel model for quality lymphedema care that has the capacity to improve clinical LEL care broadly. Our findings reveal avenues for future research exploring effective implementation of lymphedema programming and education for oncology care practitioners to improve continuity of care after cancer treatment.

## Electronic supplementary material

ESM 1 (PDF 622 kb)

ESM 2 (PDF 591 kb)

## Data Availability

The data collected during and analysed within the current study are available from the corresponding author upon reasonable request.
