# Supplementary material for: Living with leg lymphedema: developing a novel model of quality lymphedema care for cancer survivors
Source: J Cancer Surviv. 2020 Jul 26;15(1):140–50. doi: 10.1007/s11764-020-00919-2 (PMC7822774; doi:10.1007/s11764-020-00919-2)
Supplement: Supplementary file 1 — (PDF 622 kb) [file 11764_2020_919_MOESM1_ESM.pdf]

Living with leg lymphedema: developing a novel model of quality lymphedema care for cancer survivors

Catharine Bowman BHSc (Hon),[1] Devesh Oberoi PhD,[1] Lori Radke PT CLT, [2] George J. Francis MD,[3] Linda E. Carlson PhD [1]

[1] Department of Oncology, Division of Psychosocial Oncology University of Calgary, Calgary AB, Canada

[2] Department of Rehabilitation Oncology, Supportive Care South, CancerControl Alberta, AB, Canada

[3] Department of Physical Medicine and Rehabilitation, Division of Clinical Neurosciences, University of Calgary, Calgary AB, Canada

Linda E. Carlson (Corresponding Author)

Address: 2202-2 Street SW Calgary, AB Canada T2S 3C1 Email: l.carlson@ucalgary.ca

Telephone: +1 [403] 355-3209

Fax: +1 [403] 355-3206

Online Resource 1: Interview guide used for semi-structured interviews with patient participants.

Interview Guide: Lymphedema Patient Group

Diagnosis

- In what year were you diagnosed with cancer?
- In what year were you diagnosed with LE?
- How was your LE diagnosed?
  - What led to your diagnosis?
  - What signs and symptoms did you experience before your LE was diagnosed?
- How would you describe the process of receiving your diagnosis?
  - *Prompt: East, Moderate, Difficult – Why?*
  - *Prompt: Were there any issues/challenges you encountered during this process?*
  - *Prompt: Were there delays or impeding factors related to the healthcare system or yourself?*
  - *Prompt: What do you think may have improved or accelerated your diagnosis?*
  - *Prompt: What do you think would have changed if your diagnosis was more timely? Why?*

Referral and Lymphedema Information

- Were you referred to a LE clinic or Rehabilitation Oncology (AHS) for LE diagnosis or treatment?
  - If so, what services were you referred to? Can you please describe them in detail? Was the referral timely?
  - If not, why do you believe no referral was given?
- Did you have any sort of information about LE prior to your cancer treatment?
  - If yes, how did you learn about LE? What information did you have access to?
  - If not, when did you learn about LE? What resources did you access to learn more about LE?
- Did you have a conversation with your healthcare provider about LE during treatment follow-up?
  - If yes, what did you ask? What did the healthcare provider tell you? Did you speak with an oncologist, nurse or someone else?
- Did your oncology centre provide materials to learn more on LE?
  - If yes, were they in print or an online format? Did you find them helpful? Did they include a sufficient amount of information?
- How did you first hear about LE?
  - *Prompts:*
    - General Practitioner
    - Oncologist
    - Surgeon
    - Nurse, oncology
    - Nurse, other
    - Print materials within oncology clinic
    - Print materials within GP clinic
    - Education (high school, university, post-graduate or professional studies)
    - Workplace
    - Friends or Family
    - Self-educated (If so: internet, printed materials, other)
- Do you think there is a scope for improvement in information dissemination?
  - If yes, how do you think this can be improved?
  - What sources of information would you prefer to be given to you?
  - Who should provide you with this information?
- How did you find the referral process for your LE?
  - Was there any scope for improvement? If yes, how? If not, what were the components of the process you were happy with?

Treatment & Cost

- Are you currently receiving any treatment for your LE?
    - *Prompt:* From who? Who is your primary LE healthcare provider?
- If so, what type of treatment are you receiving?
    - *Prompts:*
        - Compression garments; If so, how often do you wear them?
        - Night garments
        - Lymphatic massage; If so, how often
        - Exercise; If so, what type
        - Bandaging; If so, how often
        - Diuretics
        - Anti-inflammatory drugs
- Please describe your treatment schedule (weekly)?
- Do you find your treatment(s) effective?
    - If yes, what treatment is most effective? How often do you use this treatment?
- Do you find any of your treatments ineffective?
    - If yes, what treatment is least effective? Why do you think so?
        - Do you continue to use this treatment? Why?
- Do you find the treatment(s) for your LE challenging? Do you have worries about your LE treatment?
    - If yes, what parts are the most challenging? Which are the least challenging?
    - If yes, how do you deal with those challenges?
- What do you think about the cost of these treatments?
    - Are they expensive? Are they easily affordable?
    - Does the cost affect your personal finances?
    - Can you provide an estimated cost per each treatment used?
- Are there other components to LE that are financially demanding?
    - *Prompts:* Gym, infection, CDT, MLD?
- Are you satisfied with your current treatment(s)?
    - If new treatment options became available, would you be interested in expanding upon your current regimes?
- What other kinds of treatments would you prefer if made available?
    - *Prompts:* Drug/Pharmacological, Exercise Programs, Novel Massage Techniques, Other: Please Specify

Lymphedema Knowledge

- What is your current level of understanding about LE?
- If you were to estimate the Canadian population living with LE, how many people do you think are affected? Globally?
- How did you perceive your healthcare providers' education on LE?
    - Do you think they are well-educated on this topic? Why or why not?
- Where did you receive the most knowledge on LE?
- Were you satisfied with the degree of LE knowledge you were provided with from your healthcare provider?
    - If yes, what methods were most useful for knowledge dissemination?
    - If not, how do you feel the healthcare system could improve LE knowledge for patients? For healthcare providers?
- Are you satisfied with the LE education you have thus far from your healthcare provider?
    - Are you interested in receiving more education on LE? What format would be best to present this information to you?
    - *Prompts:* Print, seminars, webpages, in-clinic information

Complications and LE Exacerbation

- Have you ever had any complications related to your LE? What kind of complications were those? How did you treatment hose complications?
    - *Prompt:* Have you ever had cellulitis?

            - If multiple occurrences, how often to have cellulitis?
            - How do you treat your cellulitis?
- Have you ever experienced cancer recurrence during you LE?
- What worsens your LE? How do you manage it?
    - *Prompts:* Temperature extremes, humidity, extended periods standing or sitting, travel; Land? Air?, stress, missing treatment appointments

Social

- Have you noted a change in your daily activities due to your LE? What kinds of changes have you noticed? Have these changes impacted your daily life? If so, please describe
- Have you noted a change in your relationships due to your LE?
    - If yes, please describe
    - *Prompts:*
        - Parent, Partner/Spouse, Children, Friends, Employers or colleagues
- What relationships are most helpful in managing your LE?
- What relationships are the most difficult when managing your LE?
- Does your LE affect your ability to work?
    - If so, please describe
- Does your LE affect your ability to travel?
    - If so, please describe
- Does your LE affect your time spent alone?
    - If so, please describe
- Does your LE affect the support you receive from others?
    - If so, please describe
- Have your familial or social obligations changed due to your LE?
    - If so, please describe
- Do you feel that others perceive you differently than before your LE? Why?

Physical

- Has your LE affected your ability to complete physically-demanding tasks? How? To what extent?
- Has your LE affected your ability to sit or stand for long periods of time?
    - If so, how? How do you accommodate this?
- Describe any physical limitations you may have due to your LE
- Do you avoid certain activities due to your LE?
    - If yes, please describe.
    - If no, do you experience any discomfort when doing certain activities? How do you manage this?
- Has your diet changed at all following your LE diagnosis? How?
- If you were to rate your overall health on a scale of 0-10 (10, most), what would your rating be?

Emotional

- Please openly describe the impact of your LE on your emotional well-being
- How do you feel towards your healthcare provider in terms of the care you have received for your LE?
    - *Prompts:*
        - Satisfied
        - Appreciative
        - Neutral
        - Frustrated
        - Angry
- Do you ever feel depressed or anxious about your LE?
    - If yes, what aspects of your LE prompt these feelings?
    - If no, what aspects of your life mitigate these feelings?
- Do you ever feel isolated due to your LE?
    - If yes, why? How do you manage this?
- Do you worry about the future of your LE? Why or why not?

- Do have fears about your LE? If yes, please describe
- Do you worry about cancer recurrence as a result of your LE? If yes, please describe
- Please describe the emotional impact of:
    - Your LE treatment
    - Any LE-related changes to your daily schedule
    - LE-related changes in finances
    - Others' perceptions of your LE
    - LE-related changes in personal relationships
    - LE-related lifestyle changes
