# Supplementary material for: Living with leg lymphedema: developing a novel model of quality lymphedema care for cancer survivors
Source: J Cancer Surviv. 2020 Jul 26;15(1):140–50. doi: 10.1007/s11764-020-00919-2 (PMC7822774; doi:10.1007/s11764-020-00919-2)
Supplement: Supplementary file 2 — (PDF 591 kb) [file 11764_2020_919_MOESM2_ESM.pdf]

Living with leg lymphedema: developing a novel model of quality lymphedema care for cancer survivors

Catharine Bowman BHSc (Hon),[1] Devesh Oberoi PhD,[1] Lori Radke PT CLT, [2] George J. Francis MD,[3] Linda E. Carlson PhD [1]

[1] Department of Oncology, Division of Psychosocial Oncology University of Calgary, Calgary AB, Canada

[2] Department of Rehabilitation Oncology, Supportive Care South, CancerControl Alberta, AB, Canada

[3] Department of Physical Medicine and Rehabilitation, Division of Clinical Neurosciences, University of Calgary, Calgary AB, Canada

Linda E. Carlson (Corresponding Author)

Address: 2202-2 Street SW Calgary, AB Canada T2S 3C1 Email: l.carlson@ucalgary.ca

Telephone: +1 [403] 355-3209

Fax: +1 [403] 355-3206

Online Resource 2: Interview guide used for semi-structured interviews with healthcare provider participants.

<u>Interview Guide: Healthcare Provider Group</u>

Referral Practices

- How many patients per month do you see (estimate) in clinic with LE-like symptoms or an official LE diagnosis?
- How many referrals do you make per month to Rehabilitation Oncology or a LE clinic
- Do you refer patients to Rehabilitation Oncology or a LE clinic who have LE-like symptoms?
- Do you refer patients who do not show symptoms, but are at-risk?
    - What are risk factors for LE?
    - What is the LE referral protocol?

Lymphedema Awareness & Knowledge

- What is your understanding of LE?
    - *Prompts: Very minimal, minimal, sufficient, well understood, very well understood*
- Were you taught about LE during your undergraduate or other professional education?
    - If so, what method of education was used? For how long do you estimate this/these lesson(s) was/were conducted?
    - If not, why do you think it was not taught? Do you feel it should have been included in your education? How did you learn about LE?
- If you were to estimate the Canadian population living with LE, how many people do you think are affected? Globally?
- Are there any resources for LE patients available within the regions? If so, please describe them in detail
- Are you able to refer patients at-risk for LE to local LE clinics? What are the referral patterns and protocols? Do you refer patients to LE clinics? What are alternative management strategies outside of the clinic?
- Please answer a few questions about LE to the best of your ability:
    - What are some causes of LE?
    - How long does it take for LE to present itself postoperatively?
    - What are some symptoms of LE?
    - How does one treat LE?
    - Are there any pharmacological treatments for LE?

Lymphedema Information Dissemination

- Do you discuss LE with your patients preoperatively? Postoperatively?
    - Please discuss why or why not
- Do you offer information on LE during appointments?
    - If so, are these materials in print or online format? Do you feel these materials have sufficient information on LE? What do you feel is the best method of teaching patients about LE?
    - If not, why do you think these materials are not provided? Do you feel it is important to make your at-risk patients aware of LE? What do you feel is the best method of teaching patients about LE?
- What type of information, if any, do you have available to outpatients re. LE?
- Do you have print or digital resources within clinic available to patients at-risk for LE?
- Do you offer educational tools or training seminars for clinic staff on LE? For patients? Do you think these tools are important to implement? What do you think is the best method to implement these tools?

Future Strategies

- Are you satisfied with the current process of patient referrals to LE clinics?

    - How could it be made easier to refer patients to local LE clinics for early-intervention?
- Would you be interested in additional education on LE? What would the best format be for your learning?
- Are you satisfied with the current state of LE knowledge dissemination for patients at-risk of developing LE? How could it be made easier to provide at-risk patients with LE-related materials?
- Do you have any other suggestions on how to improve LE-related care within the community?
